# Comparative Effects of Live and Heat-Inactivated *Enterococcus faecium* on Growth Performance and Intestinal Health in Growing Female Mink (*Neovison vison*)

**DOI:** 10.3390/vetsci13101070

**Published:** 2026-10-10

**Authors:** Guang Wang, Lin Cao, Lihua Wang

**Affiliations:** College of Animal Science and Technology, Qingdao Agricultural University, Qingdao 266109, China; 200101068@qau.edu.cn (G.W.); 13963906513@163.com (L.C.)

**Keywords:** *Enterococcus faecium*, probiotics, postbiotics, mink, growth performance, intestinal health

## Abstract

This study was conducted to evaluate the individual and combined effects of live *E. faecium* (EF) and heat-inactivated *E. faecium* (PEF) on growth performance, antioxidant capacity, immune responses, intestinal morphology and microbiota in growing female mink. A total of 120 12-week-old female mink were allocated to four dietary treatments in a 2 × 2 factorial design, with EF and PEF each supplied at 0 or 0.10%. The four diets were a basal diet (Control), basal diet + 0.10% EF (viable count > 1 × 10^8^ CFU/kg basal diet), basal diet + 0.10% PEF, and basal diet + 0.10% EF + 0.10% PEF. The feeding trial lasted 7 weeks. PEF supplementation increased final body weight and average daily gain and decreased the feed-to-gain ratio (*p* < 0.05). PEF also increased duodenal and jejunal villus height and the villus height-to-crypt depth ratio, raised jejunal secretory immunoglobulin A (SIgA), and decreased serum malondialdehyde (MDA) and jejunal mucosal IL-8, IL-1β, and TNF-α concentrations (*p* < 0.05). EF supplementation increased serum IgG and total antioxidant capacity (T-AOC) and decreased jejunal crypt depth and mucosal TNF-α concentration (*p* < 0.05). Significant interactions between EF and PEF were detected for fresh pelt length, serum IgG and T-AOC, duodenal trypsin activity, jejunal mucosal IL-1β and TNF-α, and several intestinal morphometric variables (*p* < 0.05). No evidence of synergistic effects was observed. Neither EF nor PEF significantly affected alpha diversity or the dominant phyla of the intestinal microbiota. However, the relative abundances of *Fusobacterium*, *Acinetobacter*, and *Clostridium_sensu_stricto_1* differed among treatments (*p* < 0.05). Live and heat-inactivated *E. faecium* exerted distinct effects in growing female mink. PEF showed benefits for growth performance, intestinal morphology, mucosal immunity, and oxidative status, whereas EF mainly affected IgG, T-AOC, and jejunal crypt depth. The observed interaction between EF and PEF indicates that concurrent supplementation should be evaluated cautiously, as no additive effects were detected.

## Simple Summary

*Enterococcus faecium* probiotics have been widely investigated for their potential to improve animal health and production, whereas heat-inactivated *E. faecium* preparations may provide probiotic-like benefits without requiring bacterial viability. However, the comparative effects of live and heat-inactivated *E. faecium*, particularly when administered concurrently, remain unclear in mink. This study evaluated the individual and combined effects of live *E. faecium* and heat-inactivated *E. faecium* on growth performance, antioxidant capacity, immune responses, intestinal morphology and microbiota in growing female mink. Live and heat-inactivated *E. faecium* exerted distinct effects in growing female mink, while no additive effects were observed between *E. faecium* probiotics and *E. faecium* postbiotics.

## 1. Introduction

*Enterococcus faecium* is widely used as a direct-fed microbial in animal production because of its potential beneficial effects on animal health and performance [1]. Previous studies have reported that dietary supplementation with *E. faecium* probiotics can enhance animal growth performance [2], promote nutrient utilization, enhance immune function [3,4], reduce diarrhea incidence, and alleviate pathogenic bacterial infection [3,5] in several livestock species.

Despite these potential benefits, the use of live *E. faecium* preparations faces practical and biological challenges. Viability may decline during storage and passage through the gastrointestinal tract, and some *E. faecium* strains may harbor antimicrobial-resistant determinants [6,7] or exhibit opportunistic pathogenic potential [8,9]. The overuse of antibiotics has further intensified the public health crisis related to drug resistance in *E. faecium* [10,11].

Heat-inactivated *E. faecium* preparations may retain multiple bioactive components, including organic acids, short-chain fatty acids, bacteriocins, exopolysaccharides, and other microbial products [12,13]. Such preparations may offer greater stability and a potentially favorable safety profile while retaining selected biological activities. Previous research has confirmed that postbiotics derived from lactic acid bacteria can exert probiotic-like effects [14,15,16]. Nevertheless, the effects of heat-inactivated *E. faecium* compared with live *E. faecium*, and the consequences of their combined administration, have not been well documented in mink. Therefore, this study used a 2 × 2 factorial design to evaluate the main effects of live *E. faecium*, heat-inactivated *E. faecium*, and their interaction on growth performance, serum antioxidant and immunoglobulin indices, digestive enzyme activity, jejunal mucosal immunity, intestinal morphology, and intestinal microbiota in growing female mink.

## 2. Materials and Methods

### 2.1. Experimental Materials

The *E. faecium* strain used in this study was obtained from the China General Microbiological Culture Collection Center (CGMCC accession no. 29262). Sequences of 16S rRNA gene were deposited in NCBI (accession: PP886227). The strain was activated and cultured in MRS broth at 37 °C with constant shaking (180 r/min) for 24 h. The viable count of the bacterial suspension was 3.4 × 10^9^ CFU/mL. This freshly prepared suspension was stored at 4 °C for 20 days. Its viable count was determined as 7.9× 10^8^ CFU/mL. Thus, the suspension was prepared every 20 days and served as the live probiotic preparation (EF). An aliquot of the same bacterial suspension was subjected to moist heat treatment at 121 °C for 15 min to generate the heat-inactivated preparation (PEF). No viable colonies were detected in the heat-inactivated preparation (PEF) by culture-based enumeration.

### 2.2. Experimental Design

This experiment was carried out on a commercial mink farm in Haiyang City, Shandong Province, China. A 2 × 2 factorial design was adopted, with EF and PEF each included at 0 or 0.10%. A total of 120 12-week-old female healthy mink were randomly assigned to four dietary treatments, with 15 replicate cages per treatment and two mink per cage. The treatments were basal diet without EF and PEF supplementation (Control), basal diet + 0.10% EF (EF group, viable count > 1 × 10^8^ CFU/kg basal diet), basal diet + 0.10% PEF (PEF group), basal diet + 0.10% EF + 0.10% PEF (EF + PEF group).

### 2.3. Facilities and Management

The basal diet was formulated on farm according to commercial standards for growing mink. The composition and nutritional profile of the basal diet are presented in Table 1, with ingredient composition on an as-fed basis and nutrient values on an air-dry basis. Mink were housed in double-row standard metal mesh cages (75 cm × 30 cm × 45 cm) in an open-sided shed. Each cage was equipped with a nest box (30 cm × 30 cm × 30 cm). Before each feeding, EF and PEF suspension were measured according to the experimental design and mixed into the basal diet with a hand-held blender for 3 min. Animals were fed twice daily at 6:30 and 15:30, with feed placed on the top of the cage. Clean drinking water was continuously available through drinking nipples. Each cage was also equipped with a water container for bathing and thermoregulation. The mean ambient temperature was 26.24 ± 0.05 °C, relative humidity was 65.27% ± 0.05%, and natural lighting was maintained throughout the experimental period. During the experimental period, the health status of the minks was checked twice daily, and any minks with poor health or compromised welfare were promptly removed from the study.

### 2.4. Data Collection

#### 2.4.1. Growth Performance

Individual body weights were recorded at the beginning and end of the 7-week feeding trial. Average daily gain (ADG) was calculated as the difference in body weight divided by the total number of experimental days. Feed intake was recorded for consecutive 3 d per week on a per-cage basis and average daily feed intake (ADFI) per mink was estimated. The feed-to-gain ratio (F/G) was calculated as ADFI/ADG.

#### 2.4.2. Sample Collection

At the end of the 7-week experiment, all experimental mink were subjected to carbon dioxide euthanasia conducted by professional farm technicians, since their pelts had reached commercial maturity. Cardiac puncture was performed immediately after confirmation of death. Eight blood samples (target volume, 4–5 mL each) were obtained from eligible mink per group (one sample derived from a separate cage). Serum was separated by centrifugation at 3500× *g* for 10 min at 4 °C and stored at −20 °C until analysis. Samples of duodenal contents, duodenal, jejunal, and rectal swabs were collected and stored at −80 °C for determination of digestive enzyme activities, intestinal mucosal immune markers, intestinal morphology, and microbiota profiles, respectively. Fresh pelt length (measured from the tip of the nose to the root of the tail) and fresh pelt weight were determined for the collected pelts.

#### 2.4.3. Serum Immunoglobulins and Antioxidant Indices

Serum IgG, IgA, and IgM concentrations were measured using commercial enzyme-linked immunosorbent assay (ELISA) kits (Nanjing Jiancheng Bioengineering Research Institute, Nanjing, China; Cat. Nos. H106-1-2, H108-1-2, and H109-1-2, respectively). Serum MDA, SOD, glutathione peroxidase (GSH-Px), and T-AOC were determined using commercial colorimetric kits (Nanjing Jiancheng Bioengineering Research Institute, Nanjing, China; Cat. Nos. A003-1-2, A001-1-2, A005-1-2, and A015-1-2, respectively).

#### 2.4.4. Duodenal Digestive Enzyme Activities

Duodenal contents were mixed with physiological saline at a ratio of 1:9 (*w*/*v*) and homogenized on ice. After centrifugation at 3500× *g* for 10 min at 4 °C, the supernatant was collected for determination of trypsin, lipase, and α-amylase activities using commercial assay kits (Nanjing Jiancheng Bioengineering Research Institute, Nanjing, China; Cat. Nos. A080-2-2, A054-1-1, and C016-1-1, respectively).

#### 2.4.5. Jejunal Mucosal Immune Markers

Jejunal mucosa was rinsed with sterile physiological saline, collected on ice, and homogenized with nine volumes of sterile physiological saline (*w*/*v*). Following centrifugation at 3500× *g* for 10 min at 4 °C, the supernatant was collected for measurement of IL-2, IL-6, IL-8, IL-10, IL-1β, IFN-γ, TNF-α, and SIgA using commercial ELISA kits (Nanjing Jiancheng Bioengineering Research Institute, Nanjing, China; Cat. Nos. H003-1-2, H007-1-2, H008-1-2, H009-1-2, H002-1-2, H025-1-2, H052-1-2, and H108-2-2, respectively).

#### 2.4.6. Intestinal Morphology

Duodenal segments (approximately 5 cm distal to the pylorus) and jejunal segments (approximately 10 cm distal to the duodenojejunal flexure), each 2–3 cm in length, were collected, rinsed with phosphate-buffered saline to remove luminal contents, and fixed in 4% paraformaldehyde. Samples were processed using standard histological procedures, including graded ethanol dehydration, xylene clearing, paraffin embedding, and transverse sectioning at 5 μm with a rotary microtome (HistoCore BIOCUT, Leica Biosystems, Nussloch, Germany).

Approximately 15–20 consecutive sections were cut from each intestinal segment, and three sections with complete and intact architecture were selected for H&E staining. For morphometric analysis, one section with well-preserved longitudinal villus–crypt architecture was selected from the stained sections. Three non-overlapping fields were examined under a microscope (Axio Scope.A1, Carl Zeiss, Oberkochen, Germany) at 50× magnification, representative images were captured with a digital camera (Axiocam 506 color, Carl Zeiss, Oberkochen, Germany), and morphometric analysis was performed using ZEN 2011 software (Blue edition, Carl Zeiss, Oberkochen, Germany). In each field, three well-oriented villi and three associated crypts were measured, for a total of nine villi and nine crypts per animal per intestinal segment. Villus height (VH, from the villus tip to the villus–crypt junction) and crypt depth (CD, from the crypt base to the villus–crypt junction) were recorded for each villus–crypt unit, and the villus height-to-crypt depth ratio (VH/CD) was calculated. All measurements were performed by a single observer under blinded conditions, and the multiple measurements were averaged to obtain one value per animal.

#### 2.4.7. Intestinal Microbiota

The collected rectal mucosal swabs samples were analyzed intestinal microbiota according to the previous method [17]. DNA was extracted from rectal swab samples. PCR amplification was performed using an ABI GeneAmp^®^ 9700 system (Applied Biosystems, Foster City, CA, USA). Amplified products were purified using the AxyPrep DNA Gel Extraction Kit (Axygen, Union City, CA, USA) and quantification with the QuantiFluor™-ST Blue Fluorescence Quantification System (Promega, Madison, WI, USA). Sequencing was performed on an Illumina MiSeq PE 300 platform (Illumina, San Diego, CA, USA).

### 2.5. Data Statistical Analysis

All data were assessed for normality using the Shapiro–Wilk test in SPSS 25.0 (IBM Corp., Armonk, NY, USA). The model included EF, PEF, and their interactions as fixed effects, and cage as the experimental unit. When a significant interaction was detected, post-hoc comparisons of least-squares means were performed with Tukey’s adjustment for multiple testing. Statistical significance was declared at *p* < 0.05. Non-repetitive sequences were clustered into OTUs at 97% similarity, producing representative sequences and an OTU table. Alpha diversity, microbial composition and intergroup differences were analyzed via the I-Sanger cloud platform (Majorbio Bio-Pharm Technology Co., Ltd., Shanghai, China).

## 3. Results

### 3.1. Growth Performance

As shown in Table 2, EF supplementation did not significantly affect growth performance. In contrast, PEF supplementation increased final body weight (FBW) and ADG and decreased F/G (all *p* < 0.05). A significant EF × PEF interaction was detected for fresh pelt length (*p* < 0.05). PEF-supplemented mink had a greater fresh pelt length than control mink (*p* < 0.05), whereas no significant differences were detected among the EF, PEF, and EF + PEF groups (*p* > 0.05).

### 3.2. Serum Immunoglobulins and Antioxidant Status

The effects of EF and PEF on serum immunoglobulins and antioxidant indices are presented in Table 3. EF supplementation increased serum IgG and T-AOC concentrations (*p* < 0.05), whereas PEF supplementation decreased serum MDA concentrations without significantly affecting the other measured immunoglobulin or antioxidant indices (*p* > 0.05). Significant EF × PEF interactions were observed for serum IgG and T-AOC (*p* < 0.05). Further comparison indicated that serum IgG and T-AOC levels were significantly higher in the EF, PEF, and EF + PEF groups than in the control (*p* < 0.05), with no significant differences among these three supplemented groups (*p* > 0.05).

### 3.3. Duodenal Digestive Enzyme Activities

Neither EF nor PEF supplementation significantly affected duodenal trypsin, lipase and α-amylase activity of growing female mink (*p* > 0.05; Table 4). However, a significant EF × PEF interaction was observed for duodenal trypsin activity (*p* < 0.05). Mink in PEF group exhibited higher trypsin activity than in the control (*p* < 0.05), whereas no significant differences were detected among the EF, PEF, and EF + PEF groups (*p* > 0.05), suggesting a non-additive interaction between EF and PEF.

### 3.4. Intestinal Mucosal Immunity

The effects of EF and PEF on jejunal mucosal markers are shown in Table 5. EF supplementation decreased the jejunal mucosal TNF-α concentration (*p* < 0.05). PEF supplementation decreased IL-8, IL-1β, and TNF-α concentrations (*p* < 0.05), and increased SIgA content (*p* < 0.05). Significant EF × PEF interactions were observed for IL-1β and TNF-α (*p* < 0.05). Compared to the control, the EF and PEF groups had lower IL-1β concentration, while the EF + PEF group did not differ significantly from the control (*p* > 0.05). TNF-α concentrations were lower in all three supplemented groups than in the control (*p* < 0.05), with no difference among the supplemented groups (*p* > 0.05).

### 3.5. Intestinal Morphology

**Figure 1:**
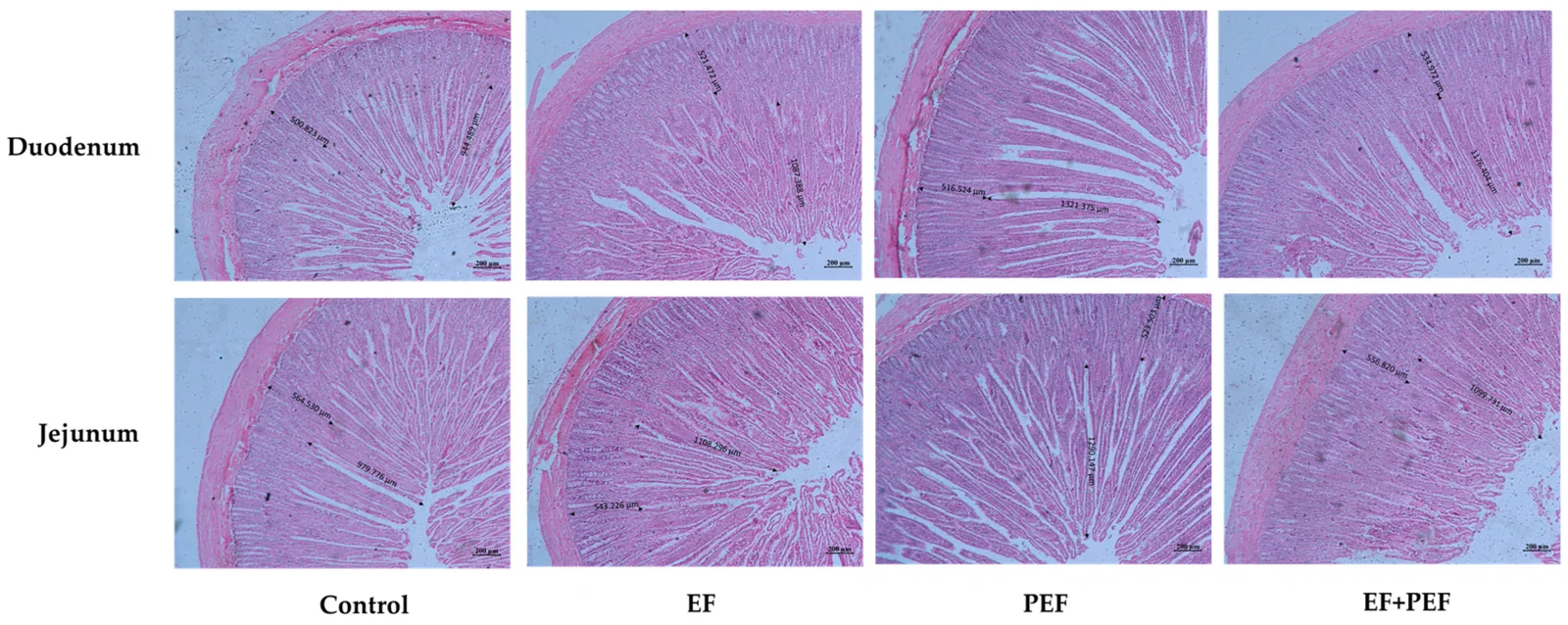
The effects of EF and PEF on duodenum and jejunum morphology in growing female mink (50×) (*n* = 8). The villus height was measured from the tip of the villus to the villus–crypt junction. The crypt depth was measured from the base of the crypt to villus–crypt junction.

The effects of EF and PEF on intestinal morphometry are presented in Table 6 and Figure 1. EF supplementation decreased jejunal CD (*p* < 0.05). PEF supplementation enhanced duodenal and jejunal VH and VH/CD (*p* < 0.05). Significant non-additive interactions between EF and PEF were detected for duodenal VH and VH/CD, as well as jejunal VH (*p* < 0.05). All supplemented groups had greater duodenal VH and VH/CD compared with the control (*p* < 0.05), with no differences among the supplemented groups (*p* > 0.05). Similarly, the EF and PEF groups exhibited higher jejunal VH compared with the control (*p* < 0.05), while the EF + PEF group did not differ from the control (*p* > 0.05).

### 3.6. Intestinal Microbiota

**Figure 2:**
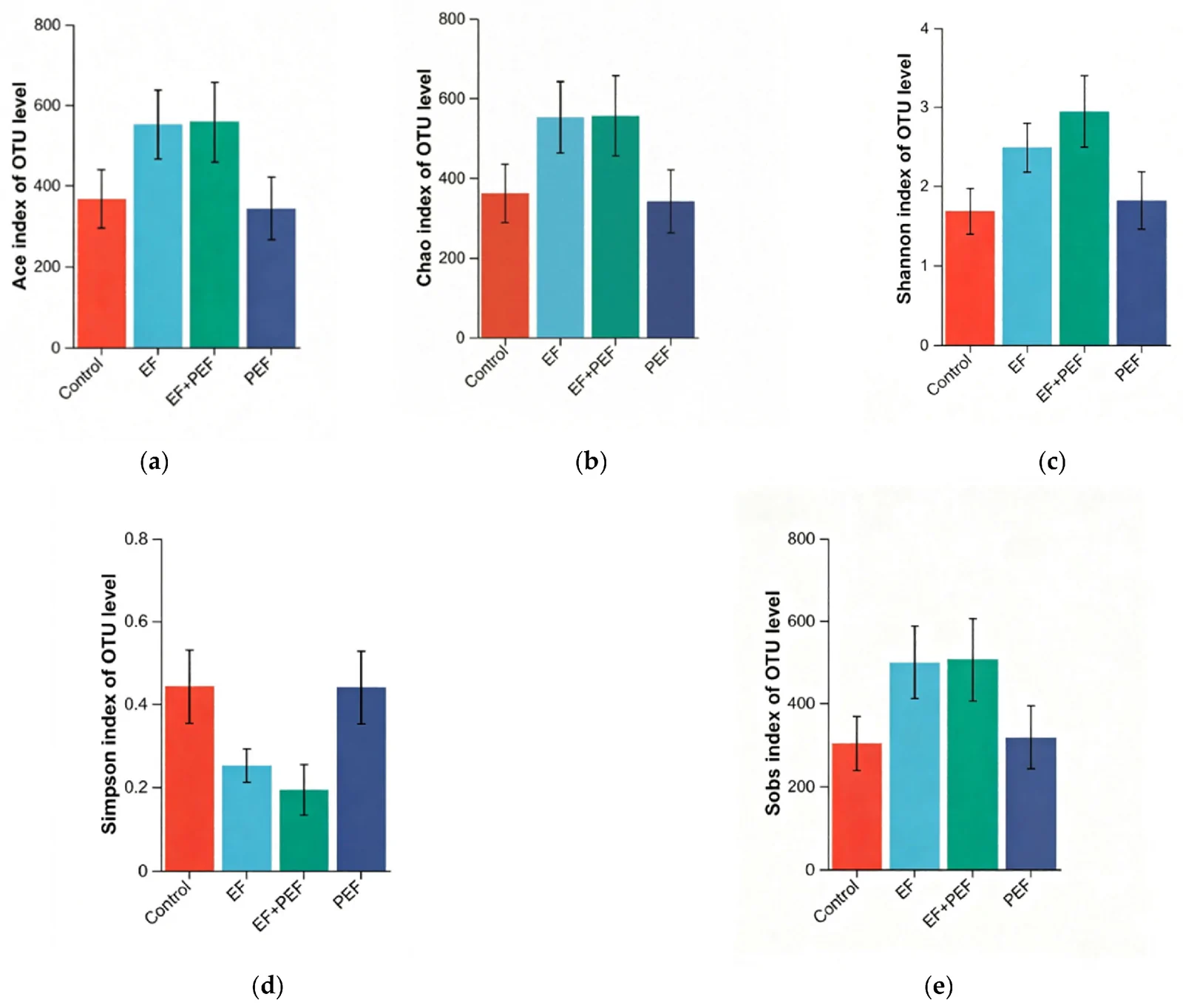
The effects of EF and PEF on Alpha diversity indices of growing female mink (*n* = 8). (**a**) Ace index; (**b**) Chao index; (**c**) Shannon index; (**d**) Simpson index; (**e**) Sobs index.

Alpha diversity was evaluated using Chao1, ACE, Sobs, Shannon, and Simpson indices. No significant differences were observed among the four groups for any of the five indices (*p* > 0.05; Figure 2), indicating that EF, PEF, and their combination did not significantly alter intestinal microbiota richness or diversity under the present experimental conditions.

**Figure 3:**
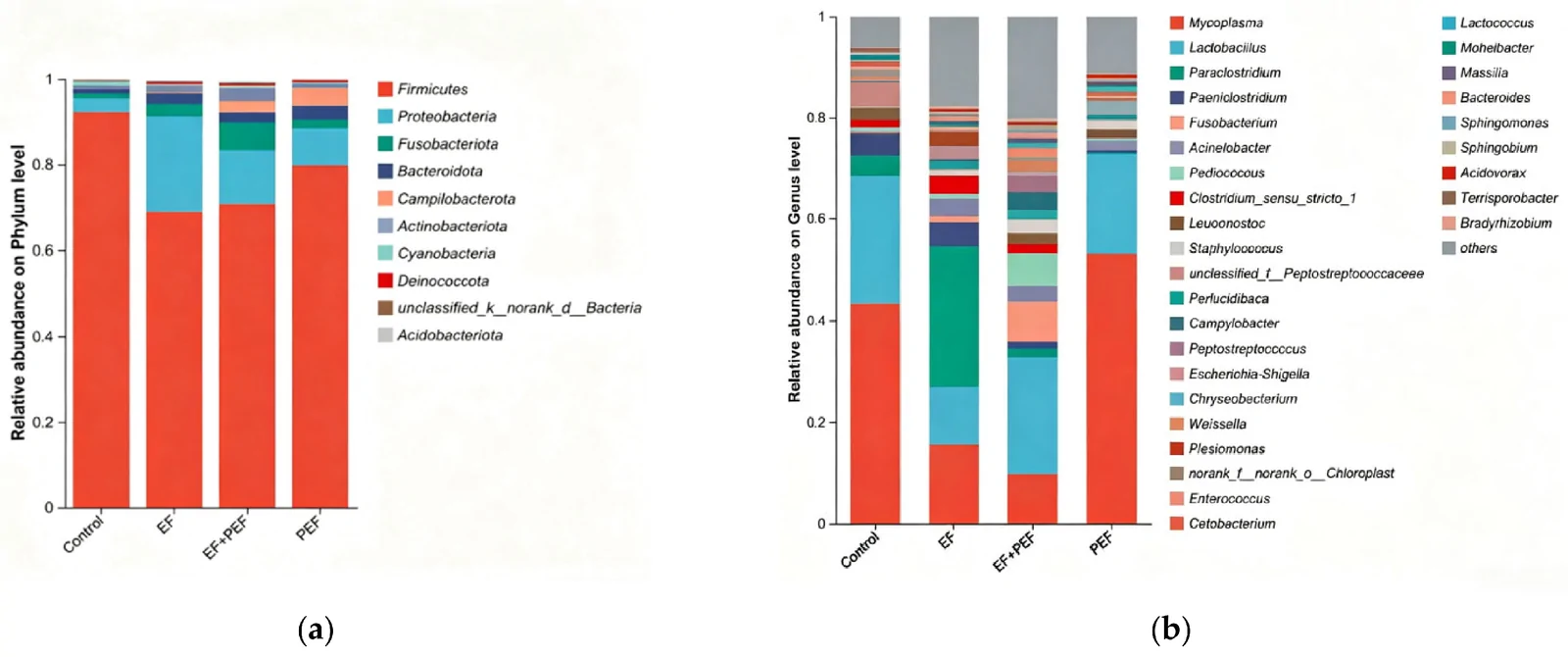
The effects of EF and PEF on the relative abundance of intestinal microbiota in growing female mink (*n* = 8). (**a**) Microflora abundance at the phylum level; (**b**) microflora abundance at the genus level.

**Figure 4:**
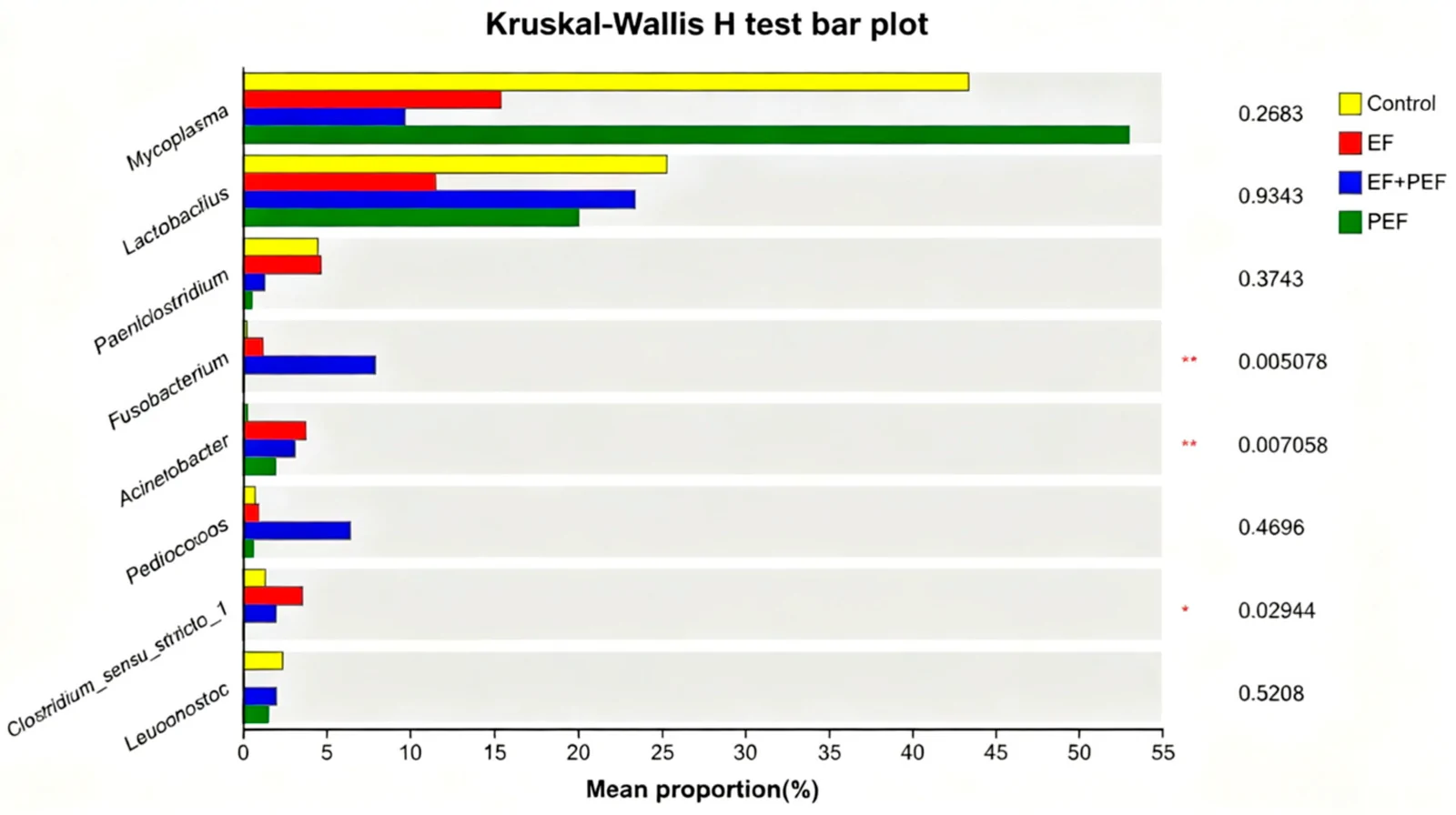
Effects of EF and PEF on difference in intestinal microbiota at genus level in growing female mink (*n* = 8). * *p* < 0.05; ** *p* < 0.01.

At the phylum level, Firmicutes, Proteobacteria, and Fusobacteriota were the three most abundant phyla in all groups and occurred in the same rank order (Firmicutes > Proteobacteria > Fusobacteriota; Figure 3a). At the genus level, the five most abundant genera in the control group were *Mycoplasma*, *Lactobacillus*, *Paraclostridium*, *Paenibacillus*, and *Fusobacterium* (Figure 3b). These genera remained predominant in the EF and EF + PEF groups, although their relative abundances were generally lower than in the control group. In the PEF group, *Mycoplasma*, *Lactobacillus*, *Paraclostridium*, and *Paenibacillus* were the predominant genera detected. Kruskal–Wallis analysis identified significant between-group differences in the relative abundances of *Fusobacterium*, *Acinetobacter*, and *Clostridium_sensu_stricto_1* (*p* < 0.05; Figure 4). *Fusobacterium* showed the highest relative abundance in the EF + PEF group, whereas *Acinetobacter* and *Clostridium_sensu_stricto_1* showed the highest relative abundance in the PEF group.

## 4. Discussion

The present study demonstrates that live and heat-inactivated *E. faecium* produced distinct responses in growing female mink. PEF supplementation was associated with improvements in growth performance, intestinal morphology, mucosal SIgA, and several inflammatory and oxidative stress markers, whereas EF primarily affected serum IgG, T-AOC, jejunal crypt depth, and mucosal TNF-α. Importantly, several EF × PEF interactions were detected, indicating that the effects of the two preparations were not consistently additive. The improvement in growth performance observed with PEF may be related to bioactive components retained after heat inactivation, including organic acids, short-chain fatty acids, bacteriocins, exopolysaccharides, and other microbial products [18,19]. These components may influence nutrient utilization and intestinal function without requiring bacterial survival or colonization. Previous studies have likewise reported beneficial effects of postbiotic preparations on animal growth and intestinal health [13,20]. In our previous study, organic acids and their derivatives in PEF constituted the largest proportion (28.7%) of the identified metabolites [21]. These metabolites are intimately involved in multiple core metabolic pathways, including central carbon metabolism, amino acid metabolism, protein synthesis, and nucleotide metabolism. Accordingly, such abundant organic acid-related metabolites within PEF may partially contribute to the improved growth performance in minks by modulating host metabolic status.

EF supplementation increased serum IgG, suggesting an effect on systemic humoral immunity. This observation is consistent with previous findings in broilers supplemented with *E. faecium* [22]. The increase in IgG may reflect immune recognition of microbial antigens and subsequent modulation of humoral immune responses [23]. PEF, in contrast, produced a more pronounced effect on mucosal immunity, as indicated by increased jejunal SIgA and reduced IL-8, IL-1β, and TNF-α concentrations. These findings suggest that the two preparations may influence different aspects of host immunity. Ren et al. [24] demonstrated in vitro that macrophages exposed to either live or heat-inactivated *E. faecium* exhibited enhanced IL-10 secretion and diminished TNF-α production. Similarly, Cui et al. [25] reported that *E. faecium* supplementation reduced pro-inflammatory cytokine expression in the jejunal mucosa of piglets. The observation that both EF and PEF reduced jejunal TNF-α indicates that both preparations exert intestinal anti-inflammatory properties, consistent with previous reports [5]. The antioxidant findings further support differential effects of EF and PEF. EF increased serum T-AOC, whereas PEF reduced serum MDA, a marker of lipid peroxidation. These results indicate that both EF and PEF can effectively enhance systemic antioxidant capacity in growing mink, which may benefit animal health and stress resilience. Previous in vitro studies have reported antioxidant activities associated with *E. faecium* and its metabolites [26,27,28], and dietary *E. faecium* has been reported to improve antioxidant status in poultry [29]. The protective effects observed here are likely mediated by antioxidant metabolites from bacterial fermentation that scavenge free radicals and upregulate endogenous antioxidant enzyme systems [26].

PEF supplementation also increased duodenal trypsin activity in the interaction analysis. Trypsin is a major proteolytic enzyme involved in the hydrolysis of dietary proteins and the generation of absorbable peptides and amino acids [30]. The higher trypsin activity observed with PEF may therefore contribute to improved nutrient utilization and feed efficiency. The interaction between EF and PEF indicates a non-additive effect of EF and PEF.

The intestinal morphological findings further offer evidence of enhanced intestinal development due to supplementation with PEF or EF. PEF increased villus height and the VH/CD ratio in both the duodenum and jejunum, whereas EF reduced jejunal crypt depth. Greater villus height and VH/CD are generally regarded as indications of improved absorptive surface area and enhanced intestinal structural integrity. Similar morphological responses have been reported that dietary supplementation with live or heat-inactivated *E. faecium* significantly increases VH and VH/CD, while decreasing CD [31,32,33]. The mechanisms responsible for these changes may involve modulation of epithelial turnover or barrier function [17,34].

The microbiota results suggest that the physiological benefits of EF and PEF supplementation were not accompanied by significant changes in alpha diversity or the dominant phyla. This finding contrasts with some studies conducted in piglets and broilers [35,36], potentially reflecting differences in host species, age, diet, husbandry conditions, bacterial strain, dosage, or sampling site. The relative stability of the dominant phyla aligns with previous descriptions of the mink gut microbiota [37,38]. Firmicutes, the predominant phylum, are a major producer of short-chain fatty acids and have been implicated in intestinal immune regulation in other animal models [39]. Conversely, Proteobacteria may trigger inflammation upon overgrowth in other studies [40]; no treatment-driven shifts in the relative abundance of dominant phyla were observed in the current study. At the genus level, Kruskal–Wallis tests revealed significant inter-group differences in the relative abundances of three genera (*Fusobacterium, Acinetobacter, and Clostridium_sensu_stricto_1*). However, no FDR or other multiple-comparison correction was applied for these genus-level microbiota comparisons; therefore, these genus-level findings should be interpreted cautiously as exploratory intergroup differences. Certain species within the genera *Fusobacterium* and *Acinetobacter* have been described as opportunistic pathogens under specific host or environmental conditions in other studies [41,42]. Nevertheless, genus-level relative abundance derived from 16S rRNA sequencing cannot distinguish pathogenic from commensal strains and does not directly indicate pathogenicity or adverse physiological outcomes in mink. Accordingly, the biological significance of the increased abundance of these genera in female mink warrants further investigation, particularly regarding their long-term impact on host health and disease susceptibility.

An important finding of the present study was the occurrence of significant EF × PEF interactions for several phenotypes, including fresh pelt length, serum IgG and T-AOC, duodenal trypsin activity, jejunal inflammatory markers, and intestinal morphometric variables. These interactions indicate that the response to combined supplementation cannot be predicted simply from the effects of either preparation alone. In particular, the absence of evidence for synergistic effects suggests that simultaneous administration should not automatically be considered advantageous. Whether the observed interactions reflect competition, altered intestinal exposure, or other biological mechanisms requires further investigation.

## 5. Conclusions

Live and heat-inactivated *E. faecium* produced distinct effects in growing female mink. EF increased serum IgG and T-AOC and reduced jejunal crypt depth, whereas PEF improved growth performance, reduced serum MDA, increased jejunal SIgA, reduced selected pro-inflammatory cytokines, and improved intestinal villus morphology. Neither preparation markedly altered alpha diversity or the dominant phyla of the intestinal microbiota, although several genera differed among treatments. Significant EF × PEF interactions were detected for multiple growth, immune, antioxidant, digestive, and intestinal morphological results. Overall, the concurrent use of live and heat-inactivated preparations should be evaluated cautiously in future studies.

## Figures and Tables

**Table 1:** Composition and nutrient levels of the basal diet.

	1–4 wk of Experiment	5–7 wk of Experiment
Ingredients		
Sea fishes	32.00	32.00
Unhatched fertilized egg	32.00	32.00
Chicken head	20.00	20.00
Extruded corn	10.00	10.00
Lard	2.00	2.00
Soybean meal	1.00	2.00
Premix ^1^	3.00	2.00
Total	100.00	100.00
Nutrient levels ^2^		
Metabolizable energy (ME, MJ/kg)	17.04	15.98
Ether extract	16.65	19.85
Crude protein	31.81	31.26
Calcium	2.47	2.59
Total phosphorus	1.59	1.64

Note: ^1^ The premix provided the following per kilogram of the diet: vitamin A 9000 IU, vitamin C 40 mg, vitamin E 20 mg, vitamin K 0.5 mg, thiamine 5 mg, riboflavin 3 mg, pyridoxine 2.5 mg, cobalamin 1 μg, vitamin D_3_ 2000 IU, nicotinic acid 20 mg, pantothenic acid 6 mg, folic acid 0.5 mg, biotin 0.5 mg, iron 30 mg, zinc 25 mg, manganese 10 mg, copper 5 mg, iodine 0.25 mg, and selenium 0.2 mg. ^2^ Dietary ME was calculated, whereas crude protein, ether extract, calcium, and total phosphorus were measured.

**Table 2:** Effects of EF and PEF on growth performance of growing female mink.

	Control	EF Group	PEF Group	EF + PEF Group	SEM	EF		PEF		*p*-Value		
						0	0.1%	0	0.1%	*P_EF_*	*P_P_*_EF_	*P*_EF*PEF_
IBW (g)	923.00	924.33	922.83	922.50	8.16	922.92	923.42	923.67	922.67	0.95	0.90	0.92
FBW (g)	1351.07	1402.86	1418.21	1445.89	25.88	1384.64	1424.38	1376.96	1432.05	0.13	0.04	0.64
ADG (g)	8.81	9.75	10.08	10.67	0.48	9.44	10.21	9.28	10.37	0.12	0.026	0.73
ADFI (g)	213.02	202.42	201.07	202.82	3.44	207.05	202.62	207.72	201.95	0.20	0.10	0.08
F/G	24.79	21.59	20.81	19.79	1.22	22.80	20.69	23.19	20.30	0.09	0.02	0.31
Fresh pelt length (cm)	51.25 ^b^	53.88 ^ab^	54.75 ^a^	53.13 ^ab^	0.91	53.00	53.50	52.56	53.94	0.60	0.16	0.04
Fresh pelt weight (g)	400.77	421.11	434.89	443.37	21.83	410.94	439.13	417.83	432.24	0.21	0.52	0.79

IBW = Initial body weight; FBW = Final body weight; ADG = Average daily gain; ADFI = Average daily feed intake; F/G = Feed-to-gain ratio. When the interaction effect was significant (*p* < 0.05), multiple comparisons were performed using Tukey’s adjustment. Means within the same row with different superscripts differed significantly (*p* < 0.05).

**Table 3:** Effects of EF and PEF on serum immunoglobulins and antioxidant indices in growing female mink.

	Control	EF Group	PEF Group	EF + PEF Group	SEM	EF		PEF		*p*-Value		
						0	0.1%	0	0.1%	*P*_EF_	*P_P_*_EF_	*P*_EF*PEF_
IgA (μg/mL)	59.61	60.57	58.41	57.00	1.79	59.01	58.79	60.09	57.70	0.90	0.19	0.52
IgG (mg/mL)	6.15 ^b^	7.57 ^a^	7.10 ^a^	6.94 ^a^	0.22	6.62	7.25	6.86	7.02	0.01	0.49	0.002
IgM (μg/mL)	470.05	503.14	486.20	487.18	16.54	478.12	495.16	486.59	486.69	0.31	0.99	0.34
SOD (U/mL)	174.08	174.45	185.23	198.65	11.90	179.66	186.55	174.27	191.94	0.57	0.15	0.59
MDA (nmol/mL)	11.73	9.44	8.21	7.83	0.97	9.97	8.64	10.58	8.02	0.19	0.02	0.34
T-AOC (U/mL)	18.53 ^b^	26.79 ^a^	25.26 ^a^	25.75 ^a^	1.38	21.89	26.27	22.67	25.51	0.01	0.06	0.01
GSH-Px (U/mL)	1943.68	2153.84	2458.70	2352.65	257.69	2201.19	2253.24	2048.76	2405.68	0.84	0.19	0.55

IgG = Serum immunoglobulin G; IgA = immunoglobulin A; IgM = immunoglobulin M; SOD = superoxide dismutase; MDA = malondialdehyde; T-AOC = total antioxidant capacity; GSH-Px = glutathione peroxidase. When the interaction effect was significant (*p* < 0.05), multiple comparisons were performed using Tukey’s adjustment. Means within the same row with different superscripts differed significantly (*p* < 0.05).

**Table 4:** Effects of EF and PEF on duodenum digestive enzyme activities in growing female mink.

	Control	EF Group	PEF Group	EF + PEF Group	SEM	EF		PEF		*p*-Value		
						0	0.1%	0	0.1%	*P*_EF_	*P_P_*_EF_	*P*_EF*PEF_
Trypsin (U/mgprot)	201.23 ^b^	290.96 ^ab^	345.56 ^a^	255.60 ^ab^	40.98	273.39	273.28	246.10	300.58	1.00	0.20	0.04
Lipase (U/gprot)	49.51	60.97	53.37	71.96	12.60	51.44	66.46	55.24	62.66	0.24	0.56	0.78
Alpha-amylase (U/mgprot)	1.59	1.60	1.64	1.67	0.20	1.62	1.64	1.60	1.66	0.92	0.76	0.96

When the interaction effect was significant (*p* < 0.05), multiple comparisons were performed using Tukey’s adjustment. Means within the same row with different superscripts differed significantly (*p* < 0.05).

**Table 5:** Effects of EF and PEF on jejunal mucosal immune status in growing female mink.

	Control	EF Group	PEF Group	EF + PEF Group	SEM	EF		PEF		*p*-Value		
						0	0.1%	0	0.1%	*P*_EF_	*P_P_*_EF_	*P*_EF*PEF_
IL-2 (g/g)	3.25	3.14	2.92	2.85	0.21	3.09	3.00	3.20	2.89	0.67	0.15	0.92
IL-6 (pg/g)	311.1	302.8	321.5	322.3	23.8	316.3	312.5	306.9	321.9	0.88	0.54	0.85
IL-8 (ng/g)	1.26	1.11	0.98	1.06	0.74	1.12	1.09	1.19	1.02	0.65	0.04	0.13
SIgA (µg/g)	24.85	26.16	35.32	28.63	2.00	30.08	27.90	25.51	32.47	0.30	0.01	0.11
IL-10 (pg/g)	793.5	702.6	877.9	771.9	51.7	835.7	737.3	748.1	824.9	0.08	0.16	0.89
IL-1β (ng/g)	3.48 ^a^	2.64 ^bc^	2.09 ^c^	2.90 ^ab^	0.22	2.78	2.77	3.06	2.50	0.96	0.03	0.003
IFN-γ (ng/g)	9.61	9.16	7.48	10.31	1.34	8.55	9.74	9.39	8.89	0.39	0.72	0.24
TNF-α (ng/g)	8.04 ^a^	6.91 ^b^	6.93 ^b^	6.89 ^b^	0.17	7.49	6.90	7.48	6.91	0.004	0.01	0.01

IL-2 = interleukin-2; IL-6 = interleukin-6; IL-8 = interleukin-8; IL-10 = interleukin-10; IL-1β = interleukin-1β; IFN-γ = interferon-γ; TNF-α = tumor necrosis factor-α; SIgA = secretory immunoglobulin A. When the interaction effect was significant (*p* < 0.05), multiple comparisons were performed using Tukey’s adjustment. Means within the same row with different superscripts differed significantly (*p* < 0.05).

**Table 6:** Effects of EF and PEF on intestinal morphology in growing female mink.

	Control	EF Group	PEF Group	EF + PEF Group	SEM	EF		PEF		*p*-Value		
						0	0.1%	0	0.1%	*P*_EF_	*P_P_*_EF_	*P*_EF*PEF_
Duodenum												
VH (μm)	1017.73 ^b^	1205.21 ^a^	1275.76 ^a^	1201.03 ^a^	36.72	1146.74	1203.12	1111.47	1238.40	0.14	0.002	0.002
CD (μm)	611.95	572.87	580.04	557.95	19.40	596.00	565.41	592.41	568.99	0.13	0.24	0.67
VH/CD	1.68 ^b^	1.93 ^a^	2.12 ^a^	1.97 ^a^	0.08	1.90	1.95	1.80	2.05	0.56	0.01	0.03
Jejunum												
VH (μm)	1097.42 ^b^	1197.86 ^a^	1261.36 ^a^	1176.70 ^ab^	30.15	1179.39	1187.28	1147.64	1219.03	0.80	0.03	0.005
CD (μm)	584.30	535.31	579.06	511.61	21.80	581.68	523.46	559.80	545.33	0.01	0.51	0.68
VH/CD	1.91	2.10	2.47	2.26	0.13	2.19	2.18	2.00	2.36	0.93	0.01	0.11

VH = Villus height; CD = Crypt depth; VH/CD = villus height-to-crypt ratio. When the interaction effect was significant (*p* < 0.05), multiple comparisons were performed using Tukey’s adjustment. Means within the same row with different superscripts differed significantly (*p* < 0.05).

## Data Availability

The original contributions presented in this study are included in the article. Any further inquiries should be directed to the corresponding author.
